# Developing an Adaptive Mobile Intervention to Address Risky Substance Use Among Adolescents and Emerging Adults: Usability Study

**DOI:** 10.2196/24424

**Published:** 2021-01-15

**Authors:** Lara N Coughlin, Inbal Nahum-Shani, Meredith L Philyaw-Kotov, Erin E Bonar, Mashfiqui Rabbi, Predrag Klasnja, Susan Murphy, Maureen A Walton

**Affiliations:** 1 Department of Psychiatry Addiction Center University of Michigan Ann Arbor, MI United States; 2 Injury Prevention Center University of Michigan Ann Arbor, MI United States; 3 Institute of Social Research University of Michigan Ann Arbor, MI United States; 4 Department of Statistics Harvard University Cambridge, MA United States; 5 School of Information University of Michigan Ann Arbor, MI United States; 6 Computer Science Harvard John A Paulson School of Engineering and Applied Sciences Harvard University Cambridge, MA United States

**Keywords:** mHealth, adolescents, young adults, just-in-time adaptive intervention, alcohol misuse, cannabis, mobile phone

## Abstract

**Background:**

Substance use among adolescents and emerging adults continues to be an important public health problem associated with morbidity and mortality. Mobile health (mHealth) provides a promising approach to deliver just-in-time adaptive interventions (JITAIs) to prevent escalation of use and substance use–related consequences.

**Objective:**

This pilot study aims to describe the iterative development and initial feasibility and acceptability testing of an mHealth smartphone app, called MiSARA, designed to reduce escalation in substance use.

**Methods:**

We used social media advertisements to recruit youth (n=39; aged 16-24 years, who screened positive for past-month binge drinking or recreational cannabis use) with a waiver of parental consent. Participants used the MiSARA app for 30 days, with feasibility and acceptability data reported at a 1-month follow-up. We present descriptive data regarding behavior changes over time.

**Results:**

The results show that most participants (31/39, 79%) somewhat liked the app at least, with most (29/39, 74%) rating MiSARA as 3 or more stars (out of 5). Almost all participants were comfortable with self-reporting sensitive information within the app (36/39, 92%); however, most participants also desired more interactivity (27/39, 69%). In addition, participants’ substance use declined over time, and those reporting using the app more often reported less substance use at the 1-month follow-up than those who reported using the app less often.

**Conclusions:**

The findings suggest that the MiSARA app is a promising platform for JITAI delivery, with future trials needed to optimize the timing and dose of messages and determine efficacy.

## Introduction

### Background

Advances in mobile health (mHealth) not only allow for accessible and cost-effective interventions but also offer novel opportunities for delivering personalized and adaptive interventions in the real world. A form of personalized medicine, just-in-time adaptive interventions (JITAIs) operationalize the use of real-time data collection in individualizing content and delivery of intervention strategies [1,2]. JITAIs have been developed and evaluated for a wide range of behavioral health issues (eg, cardiovascular disease [3], diabetes [3,4], mental illness [5], smoking cessation [6,7]).

mHealth approaches are lacking in the selective prevention of substance use (eg, risky use of alcohol, cannabis) among youth during this critical period when substance use is initiated and reaches peak prevalence [8-10]. For example, past-month binge drinking (eg, >4 for females or >5 drinks for males) is reported by 10.2% of youth aged 16 to 17 years, 26.2% of youth aged 18 to 20 years, and 45.4% of youth aged 21 to 25 years [11]. Such risky drinking increases the likelihood of health, academic, and social consequences and the development of alcohol use disorders [12-15] and is associated with morbidity and mortality (ie, injury, violence, suicide, overdose) [10,12-14]. Past-month use of cannabis is reported in 6.5% of adolescents and 22.1% of emerging adults [16]. The recent legalization of recreational cannabis (ages ≥21 years) in many areas heightens concern, particularly given the decreases in perceptions of risk [17,18] and effects on neuromaturational brain development (eg, compromised decision making and inhibitory control functioning) [19,20]. Furthermore, recent trends in the simultaneous use of alcohol and cannabis (defined as consumption patterns of alcohol and cannabis such that the individual reports the effects overlap), which is associated with greater consumption and consequences [21-24], highlight the urgent need for interventions to prevent risky substance use.

Critical challenges exist in developing mHealth interventions to reduce substance use among adolescents and emerging adults. Existing mHealth interventions for substance use are primarily developed for adults and/or individuals with substance use disorders (SUDs; <10% of adolescents and emerging adults) [25], which miss a larger proportion of adolescents and emerging adults with risky substance use. For example, emerging research demonstrates the benefit of relapse prevention apps (eg, A-CHESS, ChessHealth [26]) delivered after SUD treatment [27-32], underscoring the promise of mHealth tools, but they are tailored for individuals with greater readiness to change and substance use severity. In addition, these tools reinforce treatment concepts, which those with risky use have yet to receive, and include therapist support, which reduces scalability and may be unnecessary for those with lower severity substance use.

In 2015, recognizing the need for prevention-focused apps, the Society for Adolescent Health and Medicine released a health education app (including substance use) for parents of adolescents, THRIVE (Society for Adolescent Health and Medicine). Although beneficial, this app primarily consists of didactic, static information (eg, facts, conversation starters, immunization records, find a doctor), so content is not tailored in real time. In addition, a systematic review of 12 studies concluded that although mHealth shows promise to address substance use, more studies are needed to develop and test the efficacy using current platforms [29]. Furthermore, there was heterogeneity across studies in terms of length of intervention delivery, intervention dose delivered, remote delivery platform, sample severity, and content included. Therefore, research to identify optimal components of mHealth tools to reduce substance use among adolescents and emerging adults is needed.

Daily monitoring and ecological momentary assessment (EMA) studies can inform JITAIs by explaining why youth binge drink or use cannabis on some days and not others. However, prior daily monitoring and EMA studies of substance use are generally lacking among adolescents, with most conducted among college student samples [33,34]. Daily monitoring and EMA studies of college students underscore the importance of both negative affect (ie, craving and anxiety) in alcohol [35,36] and cannabis use [37,38] as well as positive affect (eg, enhancement motives) in binge drinking and cannabis use [37-39].

Adequate engagement with mHealth apps is necessary for success [1,2]; however, low engagement and attrition is common [40-42], including in those apps focusing on substance use [29]. This concern is particularly salient for not-in-treatment samples of adolescents and emerging adults who may not view their substance use as problematic (eg, lower readiness to change). Thus, engagement in mHealth tools for those not seeking behavior change is a unique challenge. Although passive data collection is rapidly advancing, self-reporting remains to be a staple for tailoring intervention delivery for substance use, as passive detection of key psychological mechanisms such as motives for use is not possible. Daily monitoring and EMA studies, including a recent meta-analysis, show modest response rates (eg, 60%-70%) [43-45], despite payments of US $2 to US $5 per day (or US $60-US $150 over a month), which creates a challenge for scalability [45]. Herein, we describe recent user-centered design work to develop the initial substance abuse research assistant (SARA) app, aimed at enhancing adolescents’ and emerging adults’ engagement with daily monitoring surveys. We then describe the acceptability and feasibility findings from our pilot study of a JITAI version of SARA, called MiSARA.

### An Overview of Prior Work to Iteratively Develop SARA

Consistent with the preparation phase of the Multiphase Optimization Strategy (MOST) framework [46], our team’s prior work [47] used an iterative process through a series of formative studies with adolescents and emerging adults to inform and refine our mHealth app (see Rabbi et al [47]). These prior studies focused on using the initial SARA app for enhancing engagement in daily assessments, which are critical for JITAI tailoring, and did not include delivery of intervention content [47]. Specifically, the SARA app integrated daily and weekly self-report surveys and tasks (eg, reaction time, spatial memory tasks) with theoretically grounded engagement strategies to improve the completion of self-report surveys with minimal financial incentive, enhancing scalability. As described in a study by Rabbi et al [47], to develop and refine SARA, we conducted (1) a web-based survey to assess user perceptions about design features, (2) focus groups to obtain in-depth qualitative feedback on the app, and (3) a preliminary microrandomized trial (MRT; n=18) to refine app features [47-49]. The iteratively refined SARA app employed gamification through a virtual aquarium environment, which displays more fish as self-reporting increases; gamification included levels (ie, unlocking fish based on completion) and small monetary incentives.

Subsequently, as described in a study by Nahum-Shani et al (unpublished data, 2021), we conducted a larger MRT (N=68) of SARA with youth reporting past-month binge drinking or cannabis use to further refine the SARA app. Using data from this MRT, we assessed the proximal effect of delivering theory-based engagement strategies, including reciprocity and nonmonetary reinforcement of daily survey completion. In the MRT, we included much lower financial incentives (eg, 30-day incentives averaged US $6.24 per participant) than prior daily monitoring studies while yielding fairly comparable rates of self-reporting (eg, 60.3% daily surveys, 75% weekly surveys). Regarding acceptability, most youth rated SARA as at least somewhat fun (76.3%) and interesting (72.9%). The findings supported the efficacy of reciprocity strategies (ie, inspirational quote delivered before daily survey prompts regardless of engagement), with participants liking the inspirational quotes. Nonmonetary reinforcement in the form of entertaining memes or graphics interchange format (gifs) images delivered after self-reporting did not appear to increase engagement, with feedback indicating a preference for rewards that are more consistent with the aquarium environment (eg, points, fish). Finally, mixed evidence was found for employing nonmonetary reinforcement in the form of personalized feedback graphs based on daily data; participants wanted their data available at all times, rather than contingent on self-reporting. Together, prior work from our team with the SARA app involving iterative refinement and analysis of the MRT set the stage for the development of the MiSARA JITAI aimed at reducing alcohol and cannabis use among at-risk adolescents and emerging adults. The goal of this study is to establish the feasibility and acceptability of the MiSARA JITAI and to explore preliminary outcomes related to behavior change.

## Methods

### Study Design and Protocol

#### Recruitment

Participants were recruited through social media advertisements (eg, Facebook, Instagram) over a 2-week period to obtain a broad sample of adolescents and emerging adults aged 16 to 24 years (n=39) who reported binge drinking or cannabis use. When users clicked the advertisement, they were redirected to the web-based screening consent. A waiver of parental consent was obtained for minor participants (aged 16-17 years), given the minimal risk of the study and the possibility that adolescents would prefer not to participate in this selective prevention study on substance use if parental consent was required. Eligible participants were invited to provide contact information for verification purposes, which consisted of a completely automated public Turing test to tell computers and humans apart (CAPTCHA), checking for duplicate internet protocol addresses and examining social media profiles. Once verified by staff, eligible participants were invited via email or text to join the longitudinal study, where they would complete a baseline survey in Qualtrics (with baseline consent embedded and a waiver of parental consent), download, and use the MiSARA app for 1 month and complete a 1-month follow-up survey about the app functionality, design and content, and preliminary outcomes. The MiSARA study was approved by the Medical School at the University of Michigan (IRBMED ID: HUM00148393).

#### Inclusion or Exclusion Criteria

Screened individuals were eligible for the study if they (1) had an iPhone to download the app (as the current version was compatible only with iOS not Android), (2) screened positive for past-month binge drinking (>4 drinks for females or >5 drinks for males) or any past-month cannabis use without a medical cannabis card, and (3) met the study verification criteria described earlier.

#### Self-Report Measures

At baseline and 1-month follow-up, we collected descriptive data [50,51] (Table 1) as well as quantity and frequency of use (eg, Alcohol Use Disorder Identification Test-Consumption [AUDIT-C], 30-day Timeline Follow Back) [52-56]. We also assessed the consequences of use (assessed separately for alcohol and cannabis) [57,58], intention, importance [59], and confidence of change [60] (ranging from 0 [not at all] to 10 [very]), perceived risk [61] (ranging from 0 [not at all risky] to 4 [extremely risky]), reasons for alcohol and/or cannabis use [62,63] (eg, coping, enhancement, social), and past-month driving under the influence of alcohol or cannabis [64] (ranging from 0 [never] to 4 [>10 times]).

Daily surveys included single-item measures of stress, mood, loneliness, free time, fun, sensation seeking, and hopefulness (ranging from 0 [not at all] to 4 [a lot]), in addition to daily tasks [65-70]. The tasks were designed to capture the acute cognitive effects of substance use in a naturalistic setting by assessing the reaction time (ie, tapping speed) and spatial memory (ie, retrace flower patterns that briefly light up). Weekly surveys, prompted every Sunday, measured behavioral intentions to use cannabis and/or alcohol in the next week [71] (0 [not at all] to 4 [very]) and quantity of alcohol and cannabis use each day in the prior week [61,72].

During the 1-month follow-up, participants were also asked acceptability questions (eg, “Is the app content appropriate for people your age?” “Overall, how would you rate the app’s appearance?” “Did the aquarium affect the amount of time you spent using the app?”). Participants were also asked about self-reported *points* earned in the app, which is a proxy measure for app engagement. The prototype MiSARA app contained a software error, which resulted in a lack of archiving daily or weekly survey data or paradata, which prevented further examination of objective engagement data.

**Table 1:** Intervention content topics by day of the week.

Day of week	Content area	Example intervention messages^a^
Sunday	Coping with stress	Taking time for yourself can help reduce the amount of stress we experience! How, if at all, could you prevent stressing? You are worth it!
Monday	Coping with negative mood	Accepting things that happen in life can be hard. Sometimes to be strong means asking for help. Talk to someone who cares or text 741-741
Tuesday	Prosocial people and activities	What’re three things that used to make you feel excited or happy? Try doing/thinking about these things when you feel bored/down. Good luck!
Wednesday	Alternatives to address motives for substance use	What’re things you used to love doing with friends that you miss? Imagine doing them again. Who has your best interests in mind?
Thursday	Alternative leisure activities	Doing fun or exciting activities can lift your mood! Try thinking of an exciting thing you’d like to do. Making a bucket list can be fun
Friday or Saturday	Tools and protective strategies to reduce substance use	Exercising’s a natural high. Try a new workout & challenge yourself to a fitness goal. Look in the mirror & say 3 things you like about you. Some take breaks between drinks, so they don’t overdo it. Remind yourself that you are the fun and you make the good time. Be true to you! Think about how much money you could save by staying away from too much partying. Sounds like a great way to have extra cash. Nice going!

^a^Although daily or weekly app data were not saved because of a software error, the intervention messages were tailored based on each participant’s daily responses and weekly reports of prior week substance use.

#### Incentives

Following app installation, participants received US $20 remuneration (eg, Amazon gift card). As a software glitch prevents the determination of survey completion, incentives were provided assuming 100% compliance with daily surveys; this along with a variable probability of reinforcement schedule could result in receipt of US $11 to US $21. Research staff contacted participants on Fridays with their weekly incentive total. Participants self-administered 1-month follow-up surveys and received US $30 in remuneration via an electronic gift card.

### MiSARA JITAI

For more information about SARA intervention development, see prior publications [47-49].

#### Development

**Figure 1:**
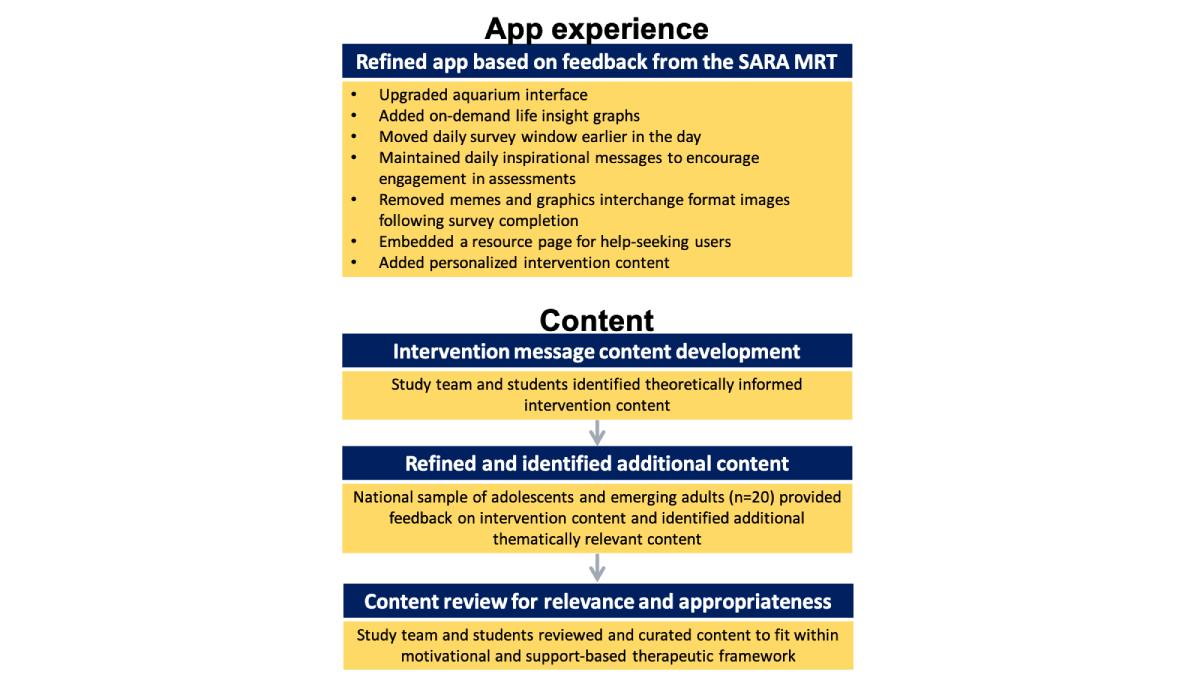
Overview of MiSARA app and content development.

**Figure 2:**
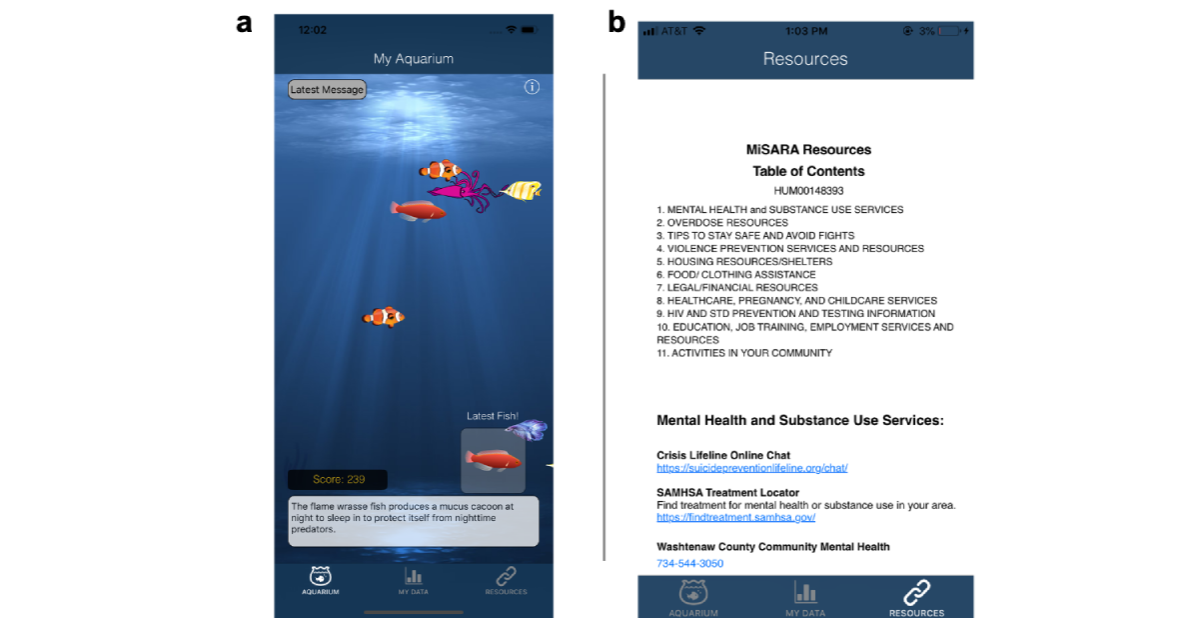
Screenshots of (a) MiSARA aquarium app environment and (b) resources page.

**Figure 3:**
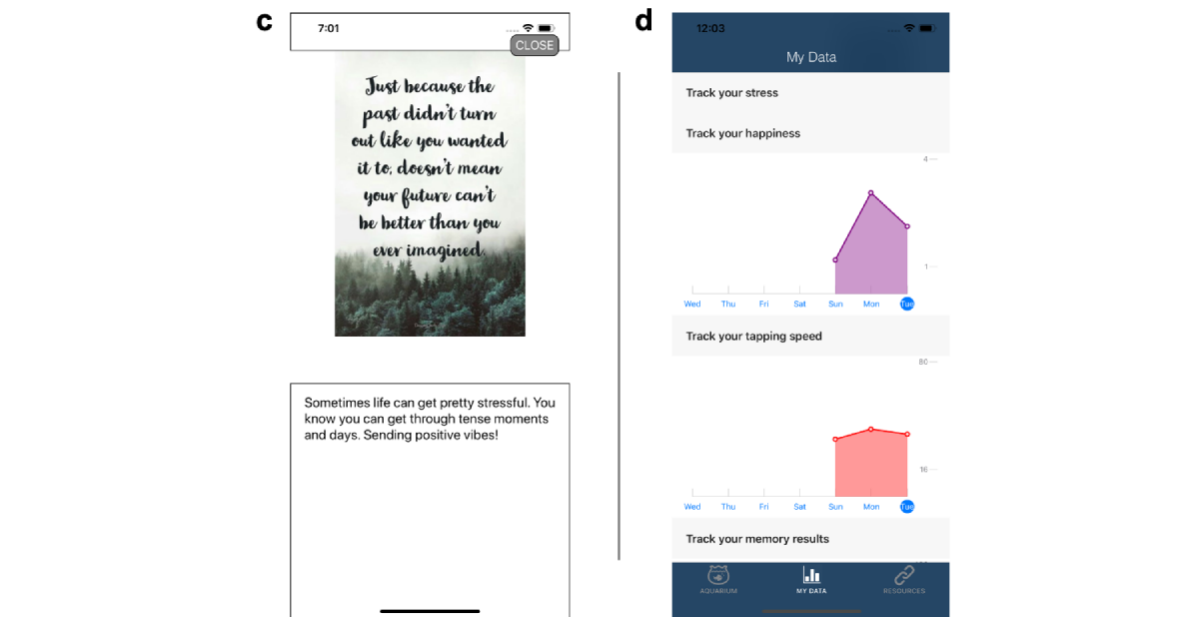
Screenshots of (c) example intervention message and (d) life insights graphs.

The MiSARA JITAI (Figure 1) was informed by SARA preliminary studies and developed for the iOS platform because most SARA participants were iPhone users and funding was insufficient for development on iOS and Android platforms. We created a more realistic aquarium interface, made personalized feedback graphs (based on within-app self-report data) available on-demand, and modified the daily survey completion window, from 6 PM to midnight in SARA and from noon to 6 PM in MiSARA. This change was made so that data from the daily survey could be used to tailor an intervention message delivered at 7 PM, potentially before a drinking event (although the timing with cannabis use is not clear). Consistent with the literature on the norm of reciprocity [73-75] and given positive findings for the daily inspirational messages (Nahum-Shani et al, unpublished data, 2021), we retained this feature, which was delivered at noon when the survey window opened. To inform refinement of the daily inspirational messages at noon, Amazon Mechanical Turk workers (n=20; mean age 21.3, SD 2.2 years; 8/20, 40% male) identified famous quotes, song lyrics, and images relevant for adolescents and emerging adults, followed by refinement by the study team to provide supporting language and by undergraduate and postgraduate students for relevance and appropriateness (eg, no images glamourizing use, no offensive jokes). We also enhanced rewards within the aquarium (eg, revamping fish artwork and delivery schedule), added a resource page with information about services and how to get help (eg, mental health, substance use), and incorporated tailored intervention content delivered at 7 PM daily (Figures 2 and 3).

Intervention content was developed and refined using an iterative participatory approach by the study team and students to fit with evidence-based intervention content (eg, motivational interviewing, mindfulness, behavioral activation) that was appealing to adolescents and emerging adults to maximize engagement. Intervention message content included supportive affirmations and tips, inspirational images to reinforce content, web links to articles (eg, 50 fun things to do without alcohol), or other web-based resources (eg, Drinkaware, YouTube meditation videos). Content areas of tailored messages varied by the day of the week (Table 1) and were tailored based on participant responses to daily surveys of factors related to substance use (eg, stress, mood, leisure activities) and to weekly surveys about substance use and motives for use [76]. For example, messages on Sunday focused on coping with stress so that participants indicating high stress on the daily stress question (“Right now, I feel stressed”) received an intervention message tailored to cope with their stress level, such as “Challenging situations can take a toll on our mental or physical health. Sometimes it helps to take a moment to ground ourselves. You’re resilient!” along with a link to a recentering mindfulness meditation YouTube video. It should be noted that if surveys were not completed, then generic content was delivered on the specified topic for that day. If a daily intervention message was delivered (based on 0.33 probability), message content was directly focused on substance use only on Wednesdays, Fridays, and Saturdays (as opposed to every day), whereas intervention message content on other days focused on upstream factors associated with use (eg, stress, mood, prosocial support, and leisure activities) to prevent fatigue and minimize unintentionally priming participants to use substances (Table 1).

#### Procedure

For a period of 30 days, participants were prompted via the MiSARA app to complete: brief (2-3 min) daily surveys, short (5-10 min) weekly surveys, and daily tasks (1-2 min each). On the basis of prior work (Nahum-Shani et al, unpublished data, 2021), each day at noon, participants received one inspirational message to encourage engagement combined with a reminder to complete the daily survey by 6 PM (ie, 7 questions, 2 tasks). On Sundays, participants were prompted to complete a weekly survey (ie, 26 questions, including past-week alcohol and cannabis use). At 7 PM each day, participants were randomized (probability=0.33) to receive (1) a tailored intervention message, (2) a fun fact on a random topic (eg, “7% of American adults believe that chocolate milk comes from brown cows. 7% doesn’t sound like a lot, but that works out to 16.4 million people”), or (3) no message, to reduce habituation to intervention content and consistent with the lower severity, not-in-treatment sample. We used the fun fact to infuse novelty so that participants would not be fatigued by too many intervention messages. To encourage engagement, participants earned points to unlock new fish for completing surveys and tasks. Following the 30-day app experience, participants completed a follow-up survey.

### Analyses

Analyses focus on the acceptability and feasibility of the MiSARA app, as this pilot study was not intended to examine MRT outcomes (eg, not powered). Open-ended, user-centered feedback about the MiSARA app experience is described. Participant ratings of feasibility and acceptability are then presented descriptively (means, SDs, proportions), which was the primary focus of this pilot study. Next, consistent with the best practices for pilot studies [76], we report (1) the association between baseline characteristics and self-reported app engagement based on zero-order correlations and a linear regression model and (2) change in preliminary outcomes from baseline to follow-up and the association between change in outcomes and self-reported app engagement.

## Results

### Recruitment Feasibility

Social media advertisements resulted in 239 participants completing the screening survey over 2 weeks, with 71 (29.7%) participants eligible. Of those eligible, 39 (55%) participated in this pilot study. No meaningful differences in age, race, or alcohol use were detected between those who did elect to participate compared with those who did not elect to participate. Those who enrolled were more likely to be female (24/39, 62%) than those who were eligible but did not enroll (11/32, 34%). In addition, those who enrolled used cannabis less frequently (mean 1.8, SD 2.1 days of cannabis use in the past 30 days) than those who were eligible but did not participate (mean 3.1, SD 2.7 days). All enrolled participants completed the 1-month follow-up.

### Sample Characteristics

Of 39 participants, 24 (62%) were female; 12 (31%) were aged 16 to 20 years; and 27 (69%) were aged 21-24 years (mean age 20.7 years, SD 2.1); 9 (23%) were identified as racial minorities and 5 (13%) were identified as Hispanic (Table 2). In the baseline survey, all participants reported alcohol use (AUDIT-C score: mean 5.9, SD 2.4), with 37 (95%) of 39 participants reporting past-month binge drinking, and 19 (49%) reporting drinking weekly or more. Of those reporting past-month cannabis use (23/39, 59%, of which 21 also reported past-month binge drinking), 11 (48%) reported using cannabis weekly or more, with 4 (17%) using daily. Overall, 36 participants reported substance use consequences in the past month, with an average of 8.5 (SD 6.7; range 0-45). Two-thirds reported driving under the effects of alcohol in the past month, and 6 (15%) of 39 participants reported driving under the effects of cannabis. As expected, given the non–treatment-seeking sample, intention and importance of reducing alcohol use was low (mean 2.4, SD 2.3 and mean 2.5, SD 1.9 respectively, on a 1-10 scale), whereas confidence in the ability to reduce alcohol was high (mean 8.3, SD 2.2 on a 1-10 scale), with a similar pattern for cannabis use, and perceived risk of regular alcohol (mean 2.5, SD 0.9 on 0-4 scale, 0 indicates no risk) and cannabis use (mean 1.3, SD 0.9 on 0-3 scale) was low. The most common motive for drinking was social, followed by enhancement (ie, to increase positive affect), with coping and other motives being the least common. Similarly, the most common reason for cannabis use was enhancement, followed by other motives and coping. The most common reason for *not* drinking or not using cannabis in the past week was not wanting to drink or use.

**Table 2:** Sample characteristics at baseline (n=39).

Demographics			Participants, n (%)	
Male sex			15 (38)	
**Age (years)^a^**				
	16-17		5 (13)	
	18-20		7 (18)	
	21-24		27 (69)	
**Race**				
	White		30 (77)	
	African American		3 (8)	
	Asian		2 (5)	
	Other		4 (10)	
	Hispanic ethnicity		5 (13)	
**Education**				
	Some high school		5 (13)	
	High school diploma or general educational development only		7 (18)	
	Some college		13 (33)	
	College graduate		14 (36)	
**Past-month substance use and consequences**				
	Binge-drinking only		16 (41)	
	Cannabis only		0 (0)	
	Alcohol and cannabis		23 (59)	
	Any substance use consequences		36 (92)	
	Drinking and driving		27 (69)	
	Cannabis and driving		6 (15)	
**Past-week top reasons for use^b^**				
	**Alcohol use (n=31)**			
		Coping		1 (3.2)
		Enhancement		11 (35.5)
		Social		15 (48.4)
		Other		4 (12.9)
	**Cannabis use (n=14)**			
		Coping		1 (7.1)
		Enhancement		10 (71.4)
		Social		0 (0.0)
		Other		3 (21.4)

^a^Mean 20.7 (SD 2.1).

^b^Past-week reasons for alcohol or cannabis use questions were only asked to those participants who reported use in the past week at baseline.

### MiSARA Acceptability

Most participants (31/39, 79%) at least somewhat liked MiSARA, with almost all reporting that the design was appealing (36/39, 92%; Table 3), but most participants also felt it was not interactive enough (27/39, 69%). Unsurprisingly, given the research context, ratings for *fun to use*, *interesting*, and *the aquarium was fun* were modest, with 27 (75%) of 36 participants indicating the aquarium itself did not influence the time spent using the app. Most importantly, the content was viewed as age-appropriate by nearly all participants (36/39, 92%). Data collection via the app was acceptable, with 36 (92%) of 39 participants agreeing that they were comfortable with self-reporting, and 28 (72%) of 39 participants agreed that they were comfortable with the app collecting passive data (such as geolocation). Although the app was easy to use (34/39, 87%) with clear instructions (35/39, 90%), 9 (23%) participants sometimes or regularly had technical problems (eg, crashing), 15 (38%) rarely had issues, and 15 (38%) never had trouble. Finally, most participants (29/39, 74%) rated the app 3 or more stars (24/39, 62% rated 3.5 or more stars; 19/39, 49% rated 4 or more stars).

Regarding appearance, qualitatively, participants reported liking the MiSARA aquarium and receiving fish, which was *nice* and *pleasant*; however, they recommended adding more animation (ie, increasing the type and number of fish, swimming speeds) and brighter colors and unique fonts. Next, participants requested greater interactivity, such as being able to feed the fish, tap on the glass, buy items or more fish, view a count of earned fish, and select fish to see maritime information at any time (not just when receiving new fish). They also recommended greater personalization (eg, type of tank or background, home page, and colors). Regarding features, participants consistently, positively commented on the life insights personal feedback graphs, requesting the addition of step counts and showing data trends for longer than a week. Similarly, they enjoyed the reaction time and spatial memory tasks but wanted more novelty in these tasks. Regarding content, participants commonly made positive comments about the *uplifting* inspirational quotes, messages, and images, links, or videos, requesting the ability to archive content, which disappeared, for later access. In addition, participants requested the ability to personalize the type of inspirational content topics or quotes, which were too *vague*. Despite all content being vetted by the study team and students, 2 people said they found some content to be *strange* or *patronizing*. Regarding functionality, although positive comments focused on the ease of the survey completion and appreciation of the reminders, a key recommendation for improving functionality was to add the ability to customize the timing of the daily survey window (eg, one person worked a mid-day shift that overlapped with the preset window). Additional suggestions included adding a second reminder to complete the survey before the end of the window, a clearer timeline of required activities and explanation of the app’s purpose, and a *pause* feature for the spatial memory task or fewer rounds (eg, to pick up a phone call). Finally, 2 people wanted more money to complete self-report surveys, and several participants noted technical issues (eg, crashing).

**Table 3:** MiSARA app acceptability (n=39).

Content area and item		Participants, n (%)^a^
**Appearance**		
	Overall	31 (79) rated average, good, excellent
	Appealing design	36 (92)
	Interactivity	26 (67) not enough 12 (31) just right 1 (3) too much
**Enjoyment**		
	Fun to use	27 (69)
	Interesting	25 (64)
	Aquarium fun to use	19 (49)
	Aquarium influenced time using app	27 (75) not at all
**Content**		
	Messages appropriate for age	36 (92)
**Data acceptability**		
	Comfort answering self-report questions	36 (92) agree or strongly agree
	Comfort with passive data collection in app	28 (72) agree or strongly agree
	Prefer self-reporting in MiSARA rather than by text or phone	32 (82)
**Technical issues**		
	Easy to use	34 (87)
	Clear app use instructions	35 (90)
	Frequency of problems	15 (38) none 15 (38) rarely 9 (23) sometimes or regularly

^a^Percent rated *3* or higher (eg, somewhat) unless otherwise indicated.

### Engagement Feasibility

Given an unanticipated software glitch, the prototype MiSARA app did not consistently save daily or weekly survey data or paradata, which prevented objective examination of app engagement. However, on the follow-up survey, participants self-reported *points* earned, a proxy for app engagement. Participants reported an average earning of 986.2 points (SD 688.0; range 0-2110; first quartile 324, third quartile 1591, median 1163; n=34; 5 missing). Self-reported app engagement (ie, points) was positively correlated with female sex (*r*=0.2) and slightly negatively correlated with age (*r*<−0.1) and negatively correlated with baseline markers of severity of substance use, including AUDIT-C score (*r*=−0.3), total number of past-month drinks (*r*=−0.4), days of binge drinking (*r*=−0.4), and days of past-month cannabis use (*r*=−0.16). In an adjusted regression model (Table 4) looking at reported app engagement as a function of age, gender, total number of past-month drinks, and total past-month cannabis use, alcohol use was the only baseline characteristic meaningfully associated with app engagement (estimate −10.5, SE 4.4; 95% CI −19.2 to −1.8), such that more alcohol use was associated with less app engagement.

**Table 4:** Linear regression of baseline predictors of app engagement.

Variable	Estimate (SE)	95% CI
Age	13.6 (53.2)	−90.7 to 117.8
Gender (referent=male)	−314.2 (216.6)	−738.7 to 110.4
Past-month alcohol use	−10.5 (4.4)	−19.2 to -1.8
Past-month cannabis use	−20.6 (23.6)	−66.9 to 25.7

### Pre-Post Changes in Preliminary Outcome

Descriptively, ratings of intention (change between follow-up and baseline (Δ_(*fu*–*bl*)=0.4) and importance (Δ_(*fu*–*bl*))=0.3) to reduce alcohol use increased slightly from baseline to 1-month follow-up, but confidence in the ability to reduce use did not increase (Δ_(*fu*–*bl*)=−0.1); in contrast, intentions (Δ_(*fu*–*bl*) =−0.4) and importance (Δ_(*fu*–*bl*)=−0.1) to reduce cannabis use decreased slightly from baseline to follow-up and confidence (Δ_(*fu*–*bl*)=0.3) increased. Regarding behaviors, all variables decreased from baseline to 1-month follow-up, including weekly alcohol (Δ_(*fu*–*bl*)=−0.8) and cannabis use (Δ_(*fu*–*bl*)=−0.2), monthly binge-drinking days (Δ_(*fu*–*bl*)=−0.4), number of past-month substance use consequences (Δ_(*fu*–*bl*)=−0.8), frequency of past-month drinking and driving (Δ_(*fu*–*bl*)=−0.8), and frequency of past-month cannabis use and driving (Δ_(*fu*–*bl*)=−0.9; Table 5). Finally, more self-reported app engagement was associated with decreases from baseline to follow-up in past-month behaviors, including total number of drinks (*r*=0.4), total number of days using cannabis (*r*=−0.1), total binge-drinking days (*r*=−0.5), substance use consequences (*r*=−0.1), episodes of drinking and driving (*r*=−0.2), and using cannabis and driving (*r*=−0.2).

**Table 5:** Baseline and follow-up substance use–related measures.

Variables		Baseline	Follow-up
**Motivation^a^, confidence^a^, and perceived risk, mean (SD)**			
	Intention to reduce alcohol	2.4 (2.3)	2.8 (2.5)
	Importance of reducing alcohol	2.3 (1.9)	2.7 (1.5)
	Confidence in ability to reduce alcohol use	8.3 (2.2)	8.2 (2.2)
	Intention to reducing cannabis use	3.2 (3.3)	2.8 (3.1)
	Importance of reducing cannabis use	2.4 (2.5)	2.3 (2.4)
	Confidence in ability to reduce cannabis use	8.6 (2.1)	8.9 (2.0)
	Perceived risk of regular alcohol use	2.5 (0.9)	2.5 (1.0)
	Perceived risk of regular cannabis use	1.3 (0.9)	1.3 (1.0)
**Substance use and consequences, mean (SD)**			
	Weekly number of alcoholic drinks	7.5 (6.1)	6.7 (6.4)
	Weekly cannabis consumption (grams)	0.5 (1.0)	0.3 (0.7)
	Number of binge-drinking episodes (past-month)	3.5 (3.3)	3.1 (3.2)
	Substance use consequences (past-month)	8.5 (6.7)	7.7 (6.8)
	Drinking and driving events (past-month)	2.5 (2.8)	1.7 (2.6)
	Cannabis and driving events (past-month)	1.6 (3.4)	0.7 (1.3)

^a^n=38 for the alcohol importance ruler and n=18 for the cannabis importance ruler. Those who stopped drinking (n=2) or using cannabis (n=1) at follow-up and therefore did not complete ratings of intention, importance, and confidence of change at follow-up were recorded as 10 for each variable.

## Discussion

### Principal Findings

Here, we provide preliminary data assessing the feasibility and acceptability of the MiSARA app for addressing a significant public health problem, risky drinking, and cannabis use among adolescents and emerging adults. The MiSARA development process was informed by behavioral theories for enhancing engagement and reducing substance misuse as well as prior work [47-49] on the assessment-focused SARA app.

In terms of feasibility, web-based recruitment using social media advertisements resulted in the recruitment of 39 participants over 2 weeks to test MiSARA, underscoring the feasibility of such methods. Although representativeness in a small pilot study cannot be determined, other larger studies support the utility of web-based recruitment approaches [77]. Perhaps because of the limited funding and constrained timeline, only 55% (39/71) of those screening positive enrolled. Those who enrolled were more likely to be female and to use cannabis less frequently than those who did not enroll. In future studies, more staffing resources, extending the completion of web-based enrollment activities beyond 2 weeks and targeting web-based recruitment toward males and those who use cannabis frequently, will likely increase enrollment rates and help ensure representativeness of the sample.

Overall, MiSARA was well-received, easy to use, and preferred over other research data collection methods (eg, website, text, telephone), with 9 out of 10 adolescents and emerging adults liking the aquarium app design. Participants were not concerned about privacy, with 9 out of 10 feeling comfortable with the self-report and 7 out of 10 being comfortable with passive data collection. Unsurprisingly, given the research purpose, ratings of the app in terms of *fun* were lower (eg, 69.2%), potentially reflecting users’ expectations regarding app-based games, which may be difficult for researchers to achieve given limited budgets and may suggest that gamification is not well-suited for research software. Another challenge for research, particularly this pilot study, was reflected in the frequency of technical software issues, including the fact that daily and weekly survey data collection was not archived. This underscores the need for strong partnerships with software developers, in addition to adequate funding for research app development and ongoing support. Despite these challenges, feasibility and acceptability data were collected for the intervention messages, which was the primary purpose of the study, given prior studies examining engagement (Nahum-Shani et al, unpublished data, 2021). Participants liked the intervention messages and viewed them as appropriate for their age. Particularly helpful feedback included the recommendation that intervention content be archived by topic so that participants could refer to it later and an additional survey completion reminder part-way through the survey window. Although participants liked the theme of the aquarium, the feedback graphs, and game-like tasks, they consistently recommended greater interactivity and personalization, which was constrained by the modest resources, consistent with formative research, in the current iteration of MiSARA. Importantly, participants wanted interactivity to evolve over time to increase novelty, raising the question of how much interactivity is needed, balanced with the costs and skills needed to develop highly interactive apps, to engender adequate engagement.

The recommendation regarding customizing the survey period underscores an interesting challenge for daily monitoring and JITAIs, namely, when to assess and deliver messages each day. In the prior version of SARA, the period of assessment was later in the day, overlapping with the time of day when participants were likely to use substances (eg, evenings). In MiSARA, the survey period was the middle of the day, with intervention messages in the evening to increase relevance to potential use periods. Another alternative would be to have a morning assessment period, assessing substance use–related factors and/or use from the prior day, followed by delivering intervention content earlier in the day. Some of these decisions will likely be influenced by the type of questions asked, the focus of the JITAI (eg, mood, substance use), and the within-day or day of the week variability in the construct (eg, binge drinking on weekend evenings).

Regarding behavior change over time, all substance use–related behaviors decreased from baseline to 1-month follow-up. Furthermore, greater app usage (as measured by self-reported points earned in the app aquarium environment) was associated with less self-reported substance use, including fewer days drinking alcohol, binge drinking, and using cannabis and fewer consequences of use and episodes of driving after drinking or using cannabis. Although these data support the potential of the MiSARA app and the motivational intervention framework, the design and scope of this single-arm pilot study, in which all participants received some intervention content (0.33 probability), precluded causal conclusions. Alternatively, these findings may reflect self-selection bias, as people motivated to make changes to their use may have used the app more often. Indeed, adolescents and emerging adults who reported using the app more had lower initial alcohol use, potentially indicating a need to broaden app features to facilitate engagement (eg, incentives, inspirational quotes) among those who report higher initial alcohol use. Regardless, these preliminary findings justify further refinement and testing of MiSARA in a fully powered trial.

### Future Research

A key future direction is the integration of passive data collection methods with self-reporting to optimize future JITAIs. For example, a recent study used passive cell phone data among 30 emerging adults over 28 days to develop a machine learning algorithm to predict nondrinking, drinking, and heavy drinking episodes (>95% accuracy), with significant predictors including features such as device usage (eg, longer keyboard press time) and movement features (eg, more changes in activities) [78,79]. The most robust predictor of drinking was time of the day (eg, evening), with the day of the week also being predictive, underscoring the timing of alcohol intervention content (eg, weekends). However, our prior studies with SARA suggest that such timing is not as useful for cannabis, which is just as likely on weekdays as weekends. Next, the Bae et al [78] study focused on identifying when drinking was already in progress, as opposed to this study, which conceptually focused on intervening before a drinking episode commences. Challenges for passive data collection include the acceptability of passive mining of cell phone data among adolescents and emerging adults (eg, in a study by Bae et al [78], 16% disabled the sensors) and feasibility across iPhone and Android devices, where privacy restrictions limit data accessibility, coupled with concerns about increased mobile phone battery use. Importantly, passive data collection is not possible to measure some important constructs that meaningfully inform tailoring of JITAIs, such as motives that are associated with use [80]. For example, Shrier et al [81] found that negative affect the day before cannabis use was a marker of next day use, as were motives. Thus, future JITAIs could integrate both passive and self-report data.

Another key direction for JITAIs is related to the timing of assessment and intervention delivery. In an ideal world, there would be an assessment of substance use patterns at the daily level (eg, mornings, afternoons, evenings, on weekdays and weekends), with assessment and intervention timing personalized over time using machine learning. However, this assessment and intervention delivery complexity requires sophisticated design and analysis methodology [82] to keep pace with the development of mHealth JITAIs.

In terms of clinical implications, if demonstrated as efficacious, the MiSARA app could be provided to youth as a universal prevention strategy, such as during a health care visit, or as part of prevention efforts at high schools or universities. Alternatively, as a selective prevention approach to alter risk trajectories and prevent the development of SUDs, the MiSARA app could be provided to adolescents and emerging adults screening positive for binge drinking or cannabis use as part of interactions in academic, primary care, or other community settings. Although our purpose was to develop an app to address primary and secondary prevention, MiSARA could also be used as an adjunct in treatment programs to provide clinicians with clinically relevant information between sessions.

### Limitations

The findings are limited by the formative nature (eg, small sample size) of this pilot study, which focused on feasibility and acceptability. Replication is required with a larger sample to determine the efficacy of intervention content on proximal substance use (eg, same day, next day, next week) and consequences from use. The limited funding for app development resulted in programming bugs, which precluded archiving of the daily data in our initial MiSARA prototype, highlighting the challenge of obtaining sufficient funding for mHealth intervention development. Thus, self-report data regarding app use (points earned) could not be verified and, despite this being a proxy for dosage, is subject to errors in recall, underscoring the need for additional testing of a refined app. Given the increasing recreational cannabis legalization, future testing should examine the efficacy of JITAIs for cannabis in states with and without such laws (eg, particularly for ages 21 years and older, who have legal access to purchase cannabis). Finally, the pilot nature of this study precludes causal determination of JITAI efficacy and necessitates future research testing of MiSARA compared with a control condition receiving only daily surveys. Nonetheless, the data collected provide clues for subsequent refinement of MiSARA and underscore the need for careful development of mHealth JITAIs.

### Conclusions

The development of real-time, real-world, personalized, and scalable JITAI approaches to reduce substance use among adolescents and emerging adults is a growing area of mHealth research. The MiSARA app is promising, as most users liked the app experience and found the content to be highly appropriate. Adolescents and emerging adult users were comfortable providing personal information on this platform and preferred it to other data collection modalities, with future refinements focusing on improving interactivity and technical functionality to sustain interest over time. Although future studies are needed to examine the efficacy of personalized JITAI content in reducing risky substance use among adolescents and emerging adults who are not in treatment, formative data showed decreases in substance use over time in the single-arm pilot study, which along with acceptability data support future testing in a full-scale trial. Consistent with the MOST framework, recommendations for JITAI development also include the use of an iterative feedback process across a series of studies to optimize efficacy.

## Abbreviations

AUDIT-C: Alcohol Use Disorder Identification Test-Consumption
EMA: ecological momentary assessment
JITAI: just-in-time adaptive intervention
SARA: substance abuse research assistant
SUD: substance use disorders
mHealth: mobile health
MOST: Multiphase Optimization Strategy
